# Vinyl halogenated fatty acids display antibacterial activity against clinical isolates of methicillin-resistant *Staphylococcus aureus*

**DOI:** 10.18103/mra.v10i7.2901

**Published:** 2022-07-31

**Authors:** David J. Sanabria-Rios, Denisse Alequin-Torres, Alenis De Jesus, Giovanni Cortes, Néstor M. Carballeira

**Affiliations:** a Faculty of Science and Technology, Department of Natural Sciences, Inter American University of Puerto Rico, Metropolitan Campus, PO Box 191293, San Juan, PR 00919, USA; b Department of Chemistry, University of Puerto Rico, Rio Piedras Campus, 17 Ave Universidad STE 1701, San Juan, PR 00925, USA

## Abstract

Methicillin-resistant *Staphylococcus aureus* is a pathogen responsible for skin and wound infections, pneumonia, and bloodstream infections. Serious attention is needed because Methicillin-resistant *S. aureus* is also resistant to many other commonly used antibiotics. This study explores the potential of vinyl halogenated fatty acids as antibacterial agents. Specifically, the total synthesis of vinyl halogenated fatty acids was performed to investigate their antibacterial activity against clinical isolates of methicillin-resistant *S. aureus*. The novel synthesis of the vinyl halogenated fatty acids was carried out by treating either 2-dodecynoic acid or 2-hexadecynoic acid with an allyl halide and 5 mol% of bis(benzonitrile)palladium (II) chloride as catalyst. Our results demonstrate that vinyl halogenated fatty acids displayed significant antibacterial activity against clinical isolates of methicillin-resistant *S. aureus* and low cytotoxicity against eukaryotic Vero Cells. Moreover, it was demonstrated that vinyl brominated fatty acids could disrupt the *S. aureus* plasma membrane and inhibit the expression of the *norB* gene.

## 1. Introduction

Methicillin-resistant *Staphylococcus aureus* (MRSA) is a pathogen responsible for skin and wound infections, pneumonia, and bloodstream infections.^1^ The Centers for Disease Control and Prevention (CDC) describes MRSA as a serious concern that requires prompt attention and rapid action.^1^ MRSA is responsible for approximately 323,700 infections in hospitals resulting in 10,600 deaths and an estimated $1.7B in total healthcare costs in the United States.^1^ Serious attention is needed because MRSA is resistant to many other commonly used antibiotics.^2,3^ This scenario becomes complicated by the lack of a big pharma pipeline to develop new compounds that target superbugs.^4^

Synthetic unsaturated fatty acids (uFA) are attractive candidates to become next-generation antibacterial agents for treating MRSA infections. Some benefits of synthetic uFA include their high antibacterial efficacy and selectivity against MRSA and their straightforward synthesis that can be performed in just a few synthetic steps.^5^ In addition, synthetic uFAs are potential antibacterial agents because they appear to have multiple mechanisms of action, making it more difficult for bacteria to develop resistance to these drugs. Synthetic uFAs can directly kill multi-drug resistant bacteria at very low concentrations (i.e., at micromolar and/or nanomolar levels).^6,7^

Many reports address the antibacterial properties of synthetic uFA against Gram-positive bacteria.^6–9^ For example, Konthikamee *et al*. reported the antibacterial activity of 2-alkynoic FA against Gram-positive bacteria, including *Staphylococcus aureus*.^8^ In 2014, Sanabria et al. performed the first study to determine the structural characteristics needed to prepare antibacterial 2-alkynoic FA.^6^ The compound that displayed the best overall antibacterial activity towards Gram-positive *S. aureus* (MIC =15.5 μg/mL) was the 2-hexadecynoic acid (2-HDA). Moreover, it was significantly effective against methicillin-resistant *S. aureus* (MRSA) (MIC = 15.6 μg/mL) and clinical isolates of MRSA (MIC = 3.9 μg/mL).^6^ In another study, Sanabria *et al*. reported that 2-HDA displayed bactericidal activity against five CIMRSA strains (MIC = 0.24–0.49 μg/mL), which were resistant to Ciprofloxacin (Cipro), indicating that 2-HDA was the most effective treatment against CIMRSA.^7^

The potential of both naturally occurring and synthetic halogenated FA against Gram-positive bacteria also have been reviewed.^10^ Specifically, it was reported that FA containing triple bonds, double bonds, and either bromine or chlorine atoms in their chemical structures are pivotal in their biological activity against Gram-positive bacteria, including *S. aureus*.^11,12^ In 2021, Latifah *et al*. reported that brominated C_18_ and C_20_ FA isolated from *Xestospongia testudinaria* displayed antibacterial activity against *Vibrio harbeyi, Vibrio parahaemolyticus, and the Vibrio alginolyticus*.^13^ The above studies emphasize the halogen moiety in the FA’s antibacterial activity. Furthermore, in another study performed by Metrangolo and Resanti, it was reported that incorporating halogen atoms into drug candidates improves the drug’s intracellular delivery because of an increment in its lipophilicity, which improves its internalization through lipid membranes and tissues.^14^ Several studies in medicinal chemistry utilize halogen substitution to improve drug pharmacokinetics.^15–17^ For this reason, halogenated FA holds promise as antibacterial agents.

A recent study reveals that the antimicrobial activity of peptoids, specifically the brominated analogs, displayed up to a 32-fold increase in the activity against *S. aureus* than its non-halogenated counterparts.^18^ Another study demonstrated that the bromination of Marula seed oil improved the inhibition activity against *S. aureus*.^19^ We recently reported the synthesis of novel vinyl halogenated FA (VHFA) and demonstrated that these FA displayed antileishmanial activity against *Leishmania infantum* promastigotes.^20^

**Scheme 1.**
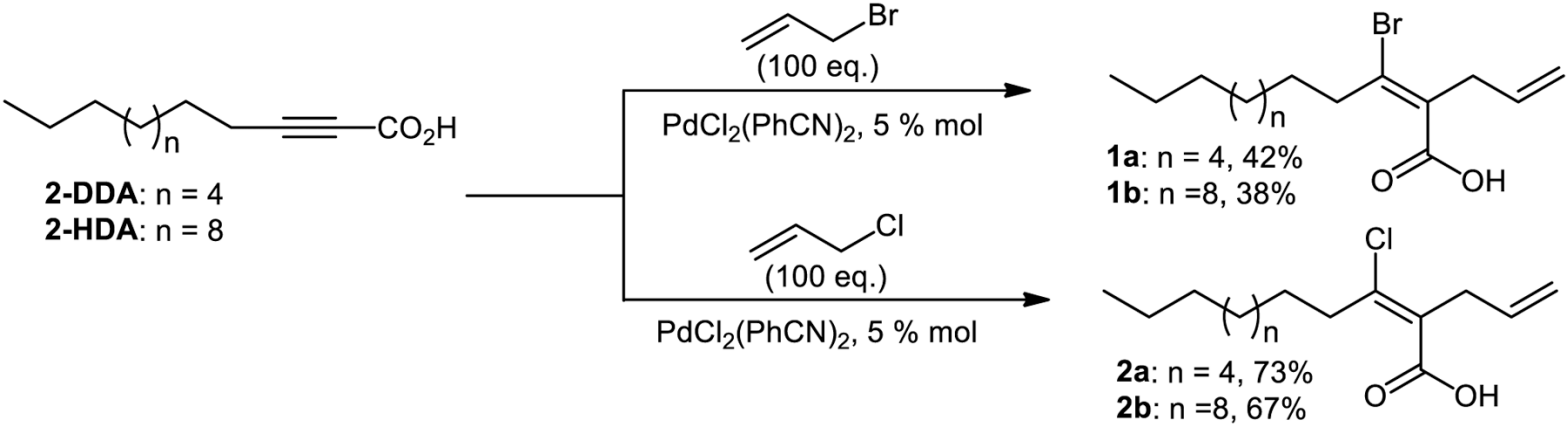
Total synthesis of the antibacterial VHFA **1a-1b** and **2a-2b**.

In this study, we explored the potential of VHFA as antibacterial agents. Herein, we also report the total synthesis of VHFA and the antibacterial activity against clinical isolates of MRSA. The antibacterial 2-allyl-3-bromo-2E-dodecenoic acid (**1a**), 2-allyl-3-chloro-2E-dodecenoic acid (**2a**), 2-allyl-3-bromo-2E-hexadecenoic acid (**1b**), and 2-allyl-3-chloro-2E-hexadecenoic acid (**2b**) were synthesized as displayed in Scheme 1.

## 2. Materials and Methods

### 2.1 Instrumentation

The glassware was flame dried under Ar or N_2_ gas and assembled with a magnetic bar, stirrer, and septum. THF was distilled over CaH and dried over Na and benzophenone before use. Hexane, ether, and ethyl acetate were purified using simple distillation. DMI and other reagents were purchased from Aldrich and Acros Organics. All reactions were analyzed by ^1^H NMR (300, 400, or 700 MHz) and ^13^C NMR (75, 100, or 176 MHz) using a Bruker DPX-300, Bruker Advance DRX-400, or Bruker Ascend Aeon 700 spectrometer. The samples were dissolved in 99.8% chloroform-d (CDCl_3_), and the solvent signals at 7.26 (^1^H) and 77.0 (^13^C) ppm were used as internal standards for hydrogen and carbon, respectively. The IR spectra were measured either on a Bruker Tensor 27 FT-IR spectrometer or a Thermo Nicolet 1S5.

### 2.2 Synthesis of vinyl halogenated fatty acids

#### 2.2.1. 2-Allyl-3-bromo-2E-dodecenoic acid (1a)

To 0.75 g (3.82 mmol) of 2-dodecynoic acid and bis(benzonitrile) palladium (II) chloride (0.073 g, 0.19 mmol, 5 mol%), was added dropwise, under a N_2_ atmosphere, 33.0 mL (380 mmol) of allyl bromide at 0 °C. The reaction was left to react for 12 h after warming up to room temperature. The crude product was filtered through Celite and the resulting solvent was removed under vacuo. The crude product was purified over silica gel by eluting with hexane and ethyl acetate (7:3) to afford pure **1a** (0.57 g, 1.80 mmol) as a yellow oil in a 42% yield. IR (neat) λ_max_: 3700–2700 (O-H), 3082, 2929, 2858, 1695 (C=O), 1651 (C=C), 1598 (C=C), 1460, 1407, 1280, 1224, 1130, 1082, 992, 915, 724, 641 (C-Br) cm^−1^. ^1^H-NMR (CDCl_3_, 300 MHz) δ (ppm) 5.86 (1H, ddt, Jt_rans_ = 17 Hz, J_cis_ = 10 Hz, J_vic_ = 6.2 Hz, H-2’), 5.11 (2H, m, H-3’), 3.32 (2H, brd, H-1’), 2.89 (2H, brt, H-4), 1.67 (2H, m, H-5), 1.28 (12H, s, H-6 to H-12), 0.91 (3H, t, J = 6.7 Hz, -CH3); ^13^C-NMR (CDCl_3_, 75 MHz) δ (ppm) 171.04 (s, C-1), 147.06 (d, C-3), 133.58 (t, C-3’), 129.35 (s, C-2), 116.37 (d, C-2’), 40.40 (t), 38.84 (t), 31.86 (t), 29.43 (t), 29.31 (t), 29.27 (t), 28.92 (t), 28.74 (t), 28.20 (t), 14.07 (q).

#### 2.2.2. 2-Allyl-3-chloro-2E-dodecenoic acid (2a)

To 0.75 g (3.82 mmol) of 2-dodecynoic acid and bis(benzonitrile)palladium(II) chloride (0.073 g, 5 mol%) was added dropwise 33.0 mL (380 mmol) of allyl chloride under N_2_ at 0 °C. The reaction mixture was stirred for an additional 12 h. The crude product was purified with silica gel column chromatography eluting with hexane and ethyl acetate (9:1) to afford **2a** (0.76 g, 0.78 mmol) as a yellow/orange oil in a 73 % yield. IR (neat) λ_max_: 3700–2700 (O-H), 3082, 2954, 2923, 2853, 1683 (C=O), 1640 (C=C), 1602 (C=C), 1456, 1437, 1405, 1281, 1225, 1123, 1039, 990, 915, 722, 669 (C-Cl) cm^−1^. ^1^H-NMR (CDCl3 300 MHz) δ (ppm) 5.85 (1H, ddt, J_trans_ = 17 Hz, J_cis_ = 10 Hz, J_vic_ = 6.1 Hz, H-2’), 5.10 (2H, m, H-3’), 3.32 (2H, brd, H-1’), 2.90 (2H, brt, H-4), 1.67 (2H, m, H-5), 1.30 (12H, s, H-6 to H-12), 0.91 (3H, t, J = 6.6 Hz, H-12).^13^C-NMR (CDCl_3_, 75 MHz) δ (ppm) 171.59 (s, C-1), 153.15 (d, C-3), 133.59 (t, C-3’), 126.65 (s, C-2), 116.10 (d, C-2’), 38.02 (t), 35.53 (t), 31.86 (t), 29.43 (t), 29.31 (t), 29.26 (t), 28.88 (t), 28.20 (t), 25.28 (t), 14.06 (q).

#### 2.2.3. 2-Allyl-3-bromo-2E-hexadecenoic acid (1b)

To 1.00 g (3.96 mmol) of 2-hexadecynoic acid and bis(benzonitrile)palladium (II) chloride (0.0759 g, 0.2 mmol, 5% mol) were added 32.2 mL (396 mmol) of allyl bromide under argon at 0 °C. The solution was stirred fro 24h. The crude reaction mixture was filtered through celite and rotoevaporated. The crude product was purified using silica gel column chromatography eluting with hexane/ether (8:2) affording **1b** (0.494 g, 1.51 mmol) as a light-yellow solid (mp 34–36 °C) in 38% yield. IR (neat) ν_max_: 3500–2500 (O-H), 2957, 2916, 2847, 1684 (C=O), 1638 (C=C), 1609, 1458, 1433, 1402, 1281, 1227, 918, 725, 640, 609 (C-Br) cm^−1^. ^1^H-NMR (CDCl_3_ 700 MHz) δ (ppm) 5.85 (1H, m, H-2’), 5.15 (1H, ddt, J_trans_ = 17.6 Hz, J_gem_ = 1.5 Hz, J_allyl_ = 1.3 Hz, H-3’trans), 5.10 (1H, ddt, J_cis_ = 10.1 Hz, J_gem_ = 1.3 Hz, J_allyl_ = 1.3 Hz, H-3’cis) 3.34 (2H, br d, H-1’), 2.99 (2H, br t, H-4), 1.67 (2H, quint, H-5), 1.30 (18H, s, H-4–15), 0.90 (3H, m, H-16); ^13^C-NMR (CDCl_3_, 176 MHz) δ (ppm) 170.73 (s, C-1), 146.94 (d, C-2), 133.31 (t, C-3’), 129.33 (s, C-3), 116.38 (d, C-2’), 40.40 (t), 38.87 (t), 36.64 (t), 31.94 (t), 29.70 (t), 29.68 (t), 29.65 (t), 29.52 (t), 29.38 (t), 29.35 (t), 28.93 (t), 28.77 (t), 22.71 (t), 14.13 (q).

#### 2.2.4. 2-Allyl-3-chloro-2E-hexadecenoic acid (2b)

To 1.00 g (3.96 mmol) of 2-nonadecynoic acid and bis(benzonitrile)palladium(II) chloride (0.0759 g, 0.2 mmol, 5% mol), were added 32.2 mL (396 mmol) of allyl chloride under a nitrogen atmosphere at 0 °C. The solution was further stirred at this temperature fro 24 h. The crude mixture was also filtered through celite and the solvent rotoevaporated. The crude product was purified using silica gel column chromatography and eluting with hexane/ethyl acetate (9:1) affording **2b** (0.992 g, 2.41 mmol) as a light-yellow solid (mp 32–34 °C) in 67% yield. IR (neat) ν_max_: 3500–2500 (O-H), 2954, 2916, 2848, 1680 (C=O), 1639 (C=C), 1607, 1462, 1445, 1408, 1281, 1231, 910, 723, 669 (C-Cl)cm^−1^. ^1^H NMR (CDCl_3_ 400 MHz) δ (ppm) 5.88 (1H, m, H-2’), 5.15 (1H, ddt, J_trans_ = 17.2 Hz, J_gem_ = 1.7 Hz, J_allyl_ = 1.6 Hz, H-3’trans), 5.10 (1H, ddt, J_cis_ = 10.1 Hz, J_gem_ = 1.8 Hz, J_allyl_ = 1.4 Hz, H-3’cis) 3.34 (2H, br d, H-1’), 2.92 (2H, br t, H-4), 1.69 (2H, quint, H-5), 1.31 (18H, s, H-4–15), 0.93 (3H, m, H-16); ^13^C NMR (CDCl_3_, 100 MHz) δ (ppm) 172.04 (s, C-1), 153.26 (d, C-2), 133.56 (t, C-3’), 126.72 (s, C-3), 116.07 (d, C-2’), 38.82 (t), 38.03 (t), 35.50 (t), 31.92 (t), 31.58 (t), 29.68 (t), 29.65 (t), 29.63 (t), 29.49 (t), 29.36 (t), 28.89 (t), 28.21 (t), 22.62 (t), 14.05 (q).

### 2.3. Bacterial strains and growth conditions

The Gram-positive *Staphylococcus aureus* (ATCC 29213) and the Gram-negative *Escherichia coli* (ATCC 25922) were obtained from the American Type Culture Collection (Manassas, VA, USA). In addition, CIMRSA strains were generously given by a community hospital in San Juan, Puerto Rico (USA). Stock cultures were preserved on blood agar (TSA with 5% sheep blood, Remel, and Oxoid Microbiology Products, Lenexa, KS). Inoculation of a single colony in 5 mL of Trypticase Soy Broth (TSB, BD Diagnostic Systems, Franklin Lakes, NJ) was carried out to prepare suspension cultures that were incubated for 16–18 h at 37 °C. Before preparing susceptibility assays, bacteria cells were resuspended in TSB and visually standardized using 0.5 McFarland standard, providing an equivalent concentration of 1.0 × 10^8^ colony-forming units (CFU)/mL.

### 2.4. Susceptibility testing

Susceptibility tests were conducted as described by Sanabria-Rios et al.^7^ Briefly, VHFA stock solutions were prepared using 100 % DMSO. Stock solutions were serially diluted with sterile TSB, and 100 μL of each dilution were transferred to a flat-bottomed microplate well preinoculated with 10 μL of TSB containing 4–5 × 10^5^ CFU. Each well was inspected using a spectrophotometer at 620 nm using a positive control well (containing the bacterial inoculated TSB but not the VHFA treatment) and a negative control well (containing sterile TSB without VHFA solution) for further comparisons. The minimum inhibitory concentration (MIC) was the concentration at which the test compound did not show turbidity in the well after incubation for 16–18 h at 37 °C.

### 2.5 Cytotoxicity assays

Vero Cells ATCC CCL-81.5 (Kidney African Green Monkey, Manassas, VA, USA) cells were cultured in DMEM (ATCC, Manassas, VA, USA) supplemented with 10% FBS (Hyclone, Waltham, MA, USA). Vero cells were seeded at a density of 6.4 × 10^6^ cells/mL (25,000 cells/well) of medium in a 96-well plate (0.2mL/well) in triplicate (N = 3). After 24 h, cells were treated at various concentrations. Controls containing 1 % DMSO were included in the experimental design for all assays. After 48 h of exposition, the medium containing the FA understudy was removed, washed with PBS 1X (Hyclone, GE Healthcare Life Sciences, UT, USA) and MTS [3-(4,5-dimethylthiazol-2-yl)-5-(3carboxy-methoxyphenyl)2-(4-sulfophenyl)-2H-tetrazolium] solution (Promega, Wisconsin, USA) was added to each well (0.2mL/well) and incubated for 2h. Absorbance was read at 490 nm with a spectrophotometer (Bio-Rad × Mark Microplate Spectrophotometer). Cell viability was quantitively assessed by the color change associated with the bio-reduction of the MTS compound. The % of viable cells was determined based on the control.

### 2.6 Methicillin-resistant S. aureus kinetic growth assays

We followed a modified version of a previously reported procedure.^7^ CIMRSA XIII strain was grown to an optical density of 1.0 (at 600 nm) and diluted 30,000-fold in TSB. A 10 μL aliquot of diluted bacteria was added to each well of a flat-bottom 96-well plate containing 100 μL of TSB with the appropriate concentration (4 × MIC) of either palmitic acid, **1a,** or Cipro. All FA dilutions contained 1% DMSO. TSB medium containing 1% DMSO was used as a control with no treatment. The plate was incubated at 37 °C for 20 h using a Thermo Scientific Varioskan Lux plate reader and then read at 600 nm.

### 2.7. Permeability studies of the HVFA on the methicillin-resistant S. aureus cell

CIMRSA XIII was grown in 5 mL of TSB to an optical density of 1.0 at 600 nm. A 45 mL sterile TSB was inoculated with 5 mL of CIMRSA XIII and treated with a VHFA stock solution to reach a final concentration of 4 × MIC. All VHFA solutions contained 1% DMSO. TSB medium containing 1% DMSO was used as a control with no treatment. Palmitic acid was used as the negative control. CIMRSA XIII cultures containing 1% DMSO and FA were centrifuged at 5,000 rpm at 4°C for 20 minutes. The resulting pellets were washed three times with 1 × PBS and resuspended with 1,000 μL of the same aqueous medium. A 10 μL of bacterial suspension was diluted with 990 μL of 1 × PBS to obtain a 1:100 bacterial suspension. To the 1:100 bacterial suspension, 1,000 μL of 1:500 of 1% fluorescein sodium salt (Sigma Aldrich, St. Louis, MO, USA) in 1 × PBS was added and incubated at 4°C for 8–10h. A 60 μL of the 1:100 bacterial suspension was fixed in a microscope slide by adding 40 μL of a solution containing 3:1 ethanolglacial acetic acid and 4% glycerol. The fixed bacteria were washed three times with 1 × PBS, and the excess of the aqueous solution was removed with a wipe. The fixed bacteria were visualized by fluorescence microscopy (Motic, AE31E Trinocular Inverted Microscope, Richmond, Canada) using a FITC filter.

### 2.8 q RT-PCR

A 100 mL of TSB was inoculated with 20 mL of TSB containing CIMRSA XIII and subsequently treated with the corresponding treatments (Cipro, **1b**, or **2b**) at their GI_50_s. A 25 mL of each cultured TSB was pelleted, and RNA was extracted using the AllPrep^®^ Bacterial DNA/RNA/Protein Kit (Qiagen, California, USA) following the manufacturer’s instructions. cDNA synthesis was prepared using the Power SYBR^™^ Green RNA-to C_T_^™^ 1-Step Kit (Applied Biosystems, CA, USA) with the following primers: 5’-ATGGAAAAGCCGTCAAGAGA-3’ (forward primer) and 5’-AACCAATGATTGTGCAAATAGC-3’ (reverse primer) according to the manufacturer’s instructions. The resulting cDNA templates were quantitatively amplified in a Step One Real-Time PCR System (Applied Biosystems, California, USA). The cycling parameters included: initial denaturation at 95°C for 4 min, followed by 40 cycles of denaturation (95°C, 45s), annealing (57°C, 45s), extension (72°C, 55s), and final extension at 72°C for 2 min. Efflux pump genes’ relative expression was calculated using the ΔΔC_T_ method, in which the amount of the desired cDNA was normalized to an endogenous reference (housekeeping gene 16S rRNA) and relative to an in vitro calibrator, is given by the variable $2^{-\Delta \Delta_{\text{CT}}}$, where C_T_ is the cycle number of the detection threshold.

## 3. Results

The synthesis of the VHFA was performed as described in the literature.^6,20,21^ Once the synthesis of VHFA **1** and **2** was carried out, we determined the antibacterial activity against CIMRSA strains, as described by Sanabria-Rios et al.^7^ Results from antibacterial activity tests are displayed in Table 1. In this study, CIMRSA XIII was selected as a bacterial model for further mechanistic studies because this strain was very resistant to Ciprofloxacin, a broad-spectrum antibiotic used to treat bacterial infections.^22^

Table 1 shows that HVFA were particularly active against Gram-positive bacteria, and no cytotoxic effect was observed in the Gram-negative *E. coli*. Also, it can be appreciated that **1b** is a 4- to 8-fold better antibacterial agent than **1a**, **2a**, and **2b** in CIMRSA XIII. It is imperative to point out that CIMRSA XIII is the most resistant MRSA strain to Cipro than the other *S. aureus* strains displayed in Table 1.^7^

To assess the cytotoxicity of the VHFA that displayed the highest inhibitory effect towards the CIMRSA strains, **1b,** and its chlorinated counterpart, **2b**, their cytotoxic effect on the growth of Vero Cells ATCC CCL-81.5 (Kidney African Green Monkey, Manassas, VA, USA) was tested using the MTS approach. The experiments were performed in triplicate (N = 3), as described below.

**Figure 1.**
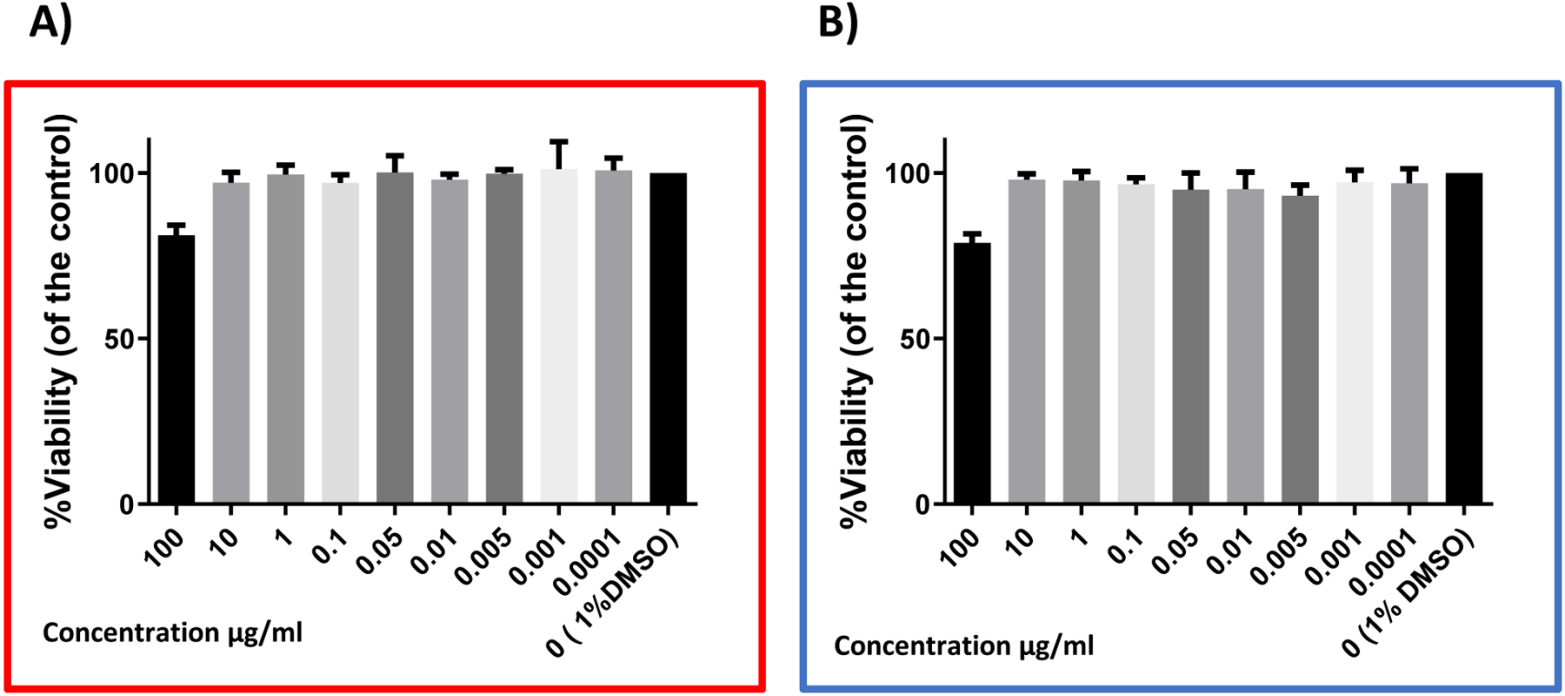
Graph bars showing the cytotoxicity of the VHFA **1b** (Figure 1A, red box) and **2b** (Figure 1B, blue box) against Vero Cells ATCC CCL-81.5. Vero cells were treated with either **1b** or **2b** at concentrations ranging from 0.0001 to 100 μg/mL. MTS reagent was added to cells after 48 h incubation. Experiments were performed in triplicates (N=3). Graphs analyses were performed with GraphPad Prism v. 6.01 (GraphPad Software

Figure 1 shows that **1b** was not cytotoxic against eukaryotic Vero Cells at a concentration ranging from 0.0001 to 10 μg/mL. Additionally, only 19% of cytotoxicity was observed when Vero Cells were treated with 100 μg/mL. Similar behavior was observed when Vero Cells were treated with 100 μg/mL of **2b**, where only 20% of cytotoxicity was obtained.

**Figure 2.**
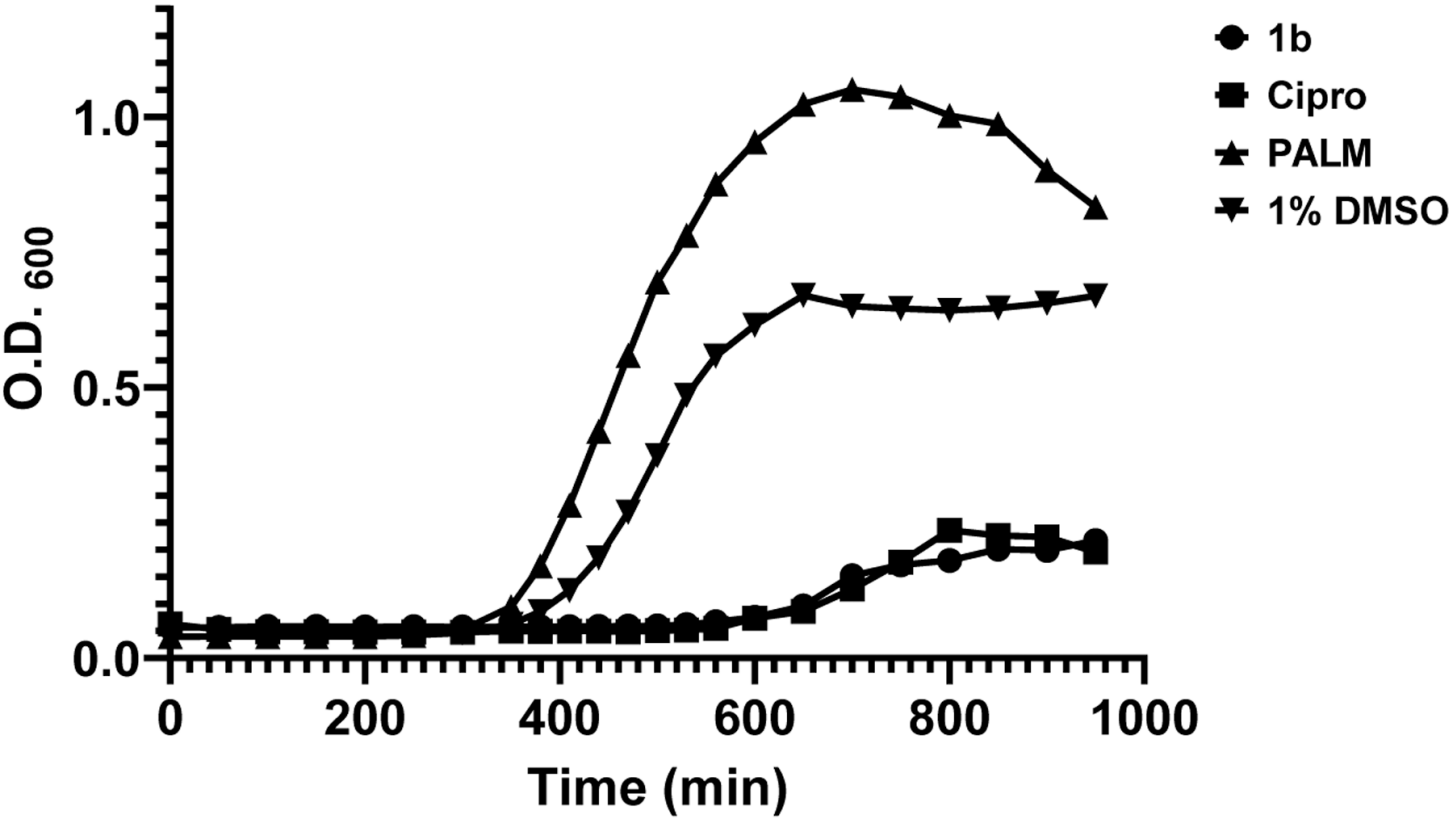
CIMRSA XIII growth curve showing its log-phase in TSB when treated with **1b** (31.2 μg/mL), Cipro (250 μg/mL), and palmitic acid (4 mg/mL). CIMRSA XIII treated with 1% DMSO was used as control. Experiments were performed in three biological replicates (N =3).

Kinetic growth assays were performed to determine whether CIMRSA XIII is adapted to metabolize **1b** (Figure 2). These assays were performed as described above.

Figure 2 reveals that **1b** displayed similar cytotoxic activity to Cipro against CIMRSA XIII. Also, it was observed that palmitic acid was less effective in inhibiting the growth of CIMRSA XIII than **1b**. The difference in O.D._600_ values between the palmitic acid and control curves can be explained by the slight turbidity when preparing TSB solutions containing palmitic acid. Both **1b** and Cipro arrested the growth of CIMRSA XIII within 600 minutes of its addition. The effectiveness of **1b** as an antibacterial agent is comparable with the Cipro curve at 31.2 μg/mL. In these assays, Cipro required a concentration of 250 μg/mL to inhibit the growth of CIMRSA XIII. Altogether, these results agree with those in Table 1 and demonstrate the potency of **1b** as an antibacterial agent in ciprofloxacin-resistant *S. aureus*.

**Figure 3.**
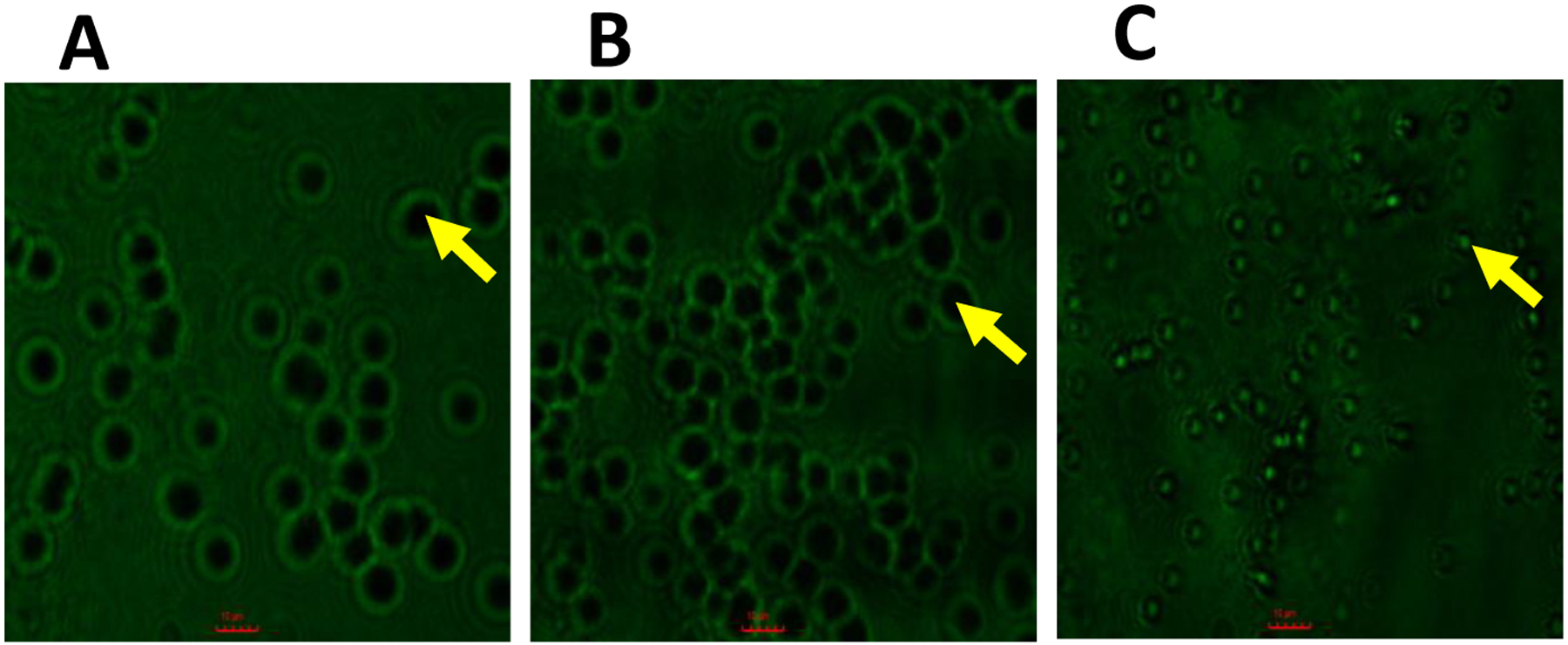
Permeability studies involving CIMRSA XIII strain. CIMRSA XIII was cultured in Tryptic Soy Broth (TSB), then stained with fluorescein disodium salt (for cell permeability; green), and visualized by fluorescence microscopy (Motic, AE31E Trinocular Inverted Microscope, Richmond, Canada). A) CIMRSA XIII in TSB containing 1% DMSO. B) CIMRSA XIII cultured in TSB containing Palmitic Acid. C) CIMRSA XIII cultured in TSB containing **1b**. CIMRSA XIII in Figures B and C were treated with the appropriate concentration of FA (i.e., 4 × MIC). Scale Bar = 10 μm.

Permeability studies on CIMRSA XIII were performed to acquire further knowledge about the mechanism of action of **1b**. The CIMRSA XIII cells used in these studies were visualized by fluorescence microscopy using a FITC filter. The results are shown in Figure 3. It can be observed that the CIMRSA XIII treated with 1% DMSO and palmitic acid did not permeabilize fluorescein inside the bacterial cell (see yellow arrows). On the other hand, the CIMRSA XIII treated with **1b** showed no well-defined membranes, the bacterium’s size was reduced, and fluorescein was observed inside the bacterium. These results suggest that **1b** kills CIMRSA XIII, possibly disrupting the cell membrane.

**Figure 4.**
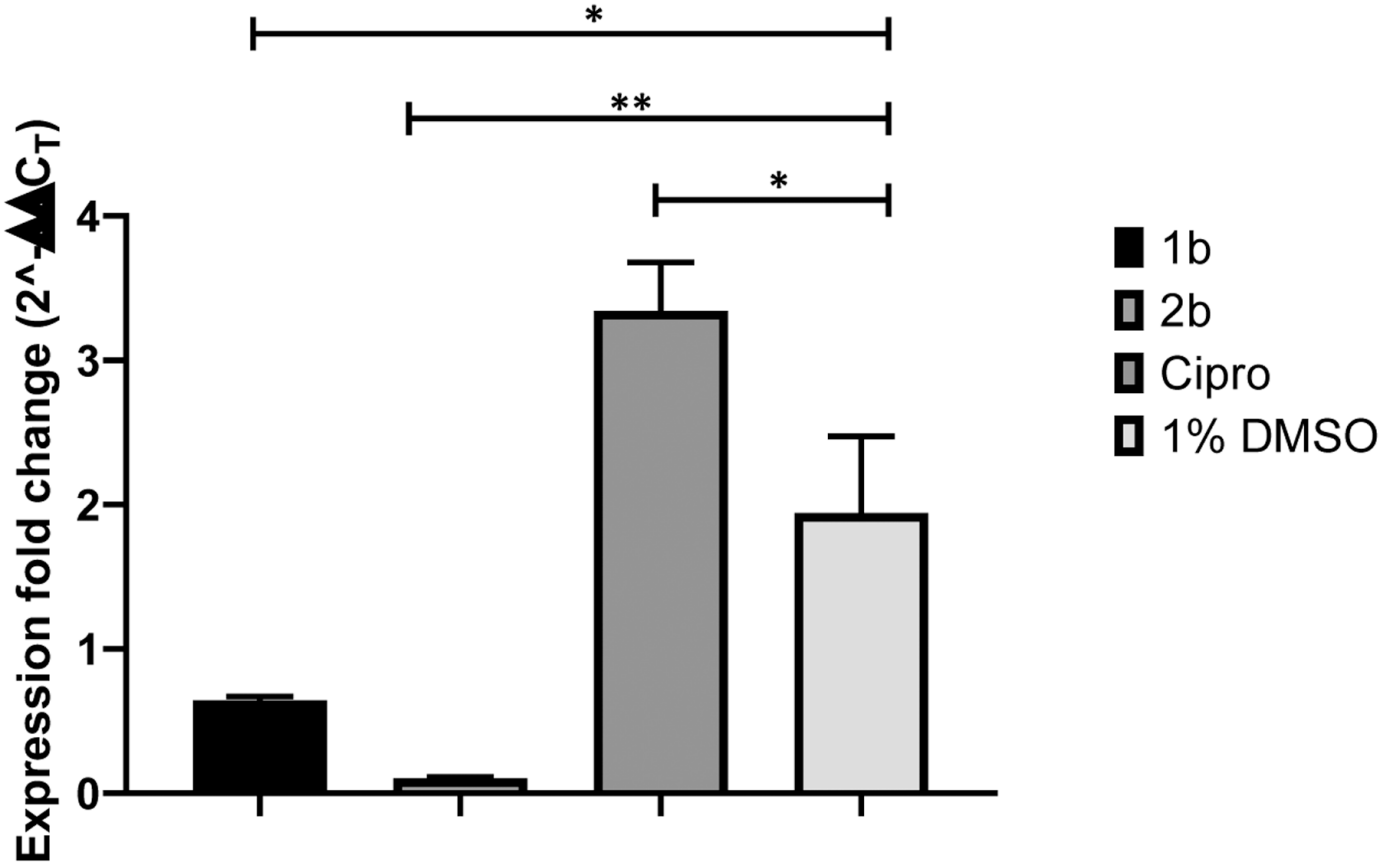
Graph bars showing the inhibitory effect of **1b** and **2b** in the expression of the *norB* gene in CIMRSA XIII. CIMRSA XIII was exposed to the treatments mentioned above at their corresponding GI_50_’s reported in Table 1. Experiments were performed in quadruplicate (N=4). Statistical analysis was performed using one-way ANOVA followed by Dunnett’s multiple comparisons test. Results were reported as mean ± SEM, and experimental groups were compared with the control group containing 1% DMSO. GP: 0.1234 (ns), 0.0332 (*), 0.0021 (**), 0.0002 (***), and <0.0001 (****).

Continuing the search to find insight into the mechanism of action of **1b** in terms of its inhibitory effect on MRSA strains, we studied its effect in expressing the *norB* gene in *S. aureus*. The *norB* gene regulates the NorB protein’s expression, an efflux pump that creates resistance to quinolones and other agents in *S. aureus*. In addition, NorB is a surface protein that provides integrity to the *S. aureus* plasma membrane.^23^ Therefore, q RT PCR was used to determine the inhibitory effect of **1b** and its chlorinated analog **2b** in the expression of the *norB* gene in *S. aureus*. Results from this study are displayed in Figure 4.

Results in Figure 4 demonstrate that both **1b** and **2b** significantly decreased the expression of *norB* when CIMRSA XIII was treated with these compounds. Another aspect observed in Figure 4 is that Cipro significantly overexpresses the *norB* gene, implying that CIMRSA is producing sufficient NorB to regulate the internalization of Cipro into the bacterial cell. Overexpression of *norB* in CIMRSA XIII is consistent with findings previously reported in the literature.^24^

## 4. Discussion

In 2020, it was determined that 2-HDA was effective in inhibiting the growth of CIMRSA strains compared with other structural isomers of this uFA.^7^ In this study, we explored the antibacterial activity of VHFA in six MRSA strains that are also resistant to Ciprofloxacin. Our results demonstrate that **1b** displayed significant antibacterial activity against the six CIMRSA strains in Table 1. Also, our results show that a cis double bond, a vinyl group, and a bromine atom are important for the antibacterial activity against CIMRSA. Another relevant structure-activity relationship (SAR) information that can be detached from Table 1 is that the presence of chlorine atoms decreases the antibacterial activity of VHFA against CIMRSA. Also, it can be observed that compounds **1a**, **1b**, **2a**, and **2b** were not active against the Gram-negative *E. coli*. This phenomenon can be due to the difference in bacterial cell wall composition. Gram-positive bacteria are absent of an outer membrane but are surrounded by thicker layers of peptidoglycan than Gram-negative bacteria.^25^ The lack of bioactivity of VHFA **1** and **2** in *E. coli* agrees with other studies reporting the selectivity of FA towards Gram-positive bacteria.^6,7^

The cytotoxic effect of HVFA **1b** and **2b** on Vero Cells’ growth was also assessed using the MTS approach to determine the selectivity of these compounds in prokaryotic cells. Results in both Table 1 and Figure 1 demonstrate that **1b** and **2b** were highly selective in *S. aureus* than in Vero Cells. Furthermore, our results demonstrated that HVFA are less cytotoxic in eukaryotic cells than 2-alkynoic FA,^6^ which highlights their potential as antibacterial agents.

Results in Figure 2 demonstrate that **1b** is a potent growth inhibitor since this compound displayed similar growth behavior to 2-HDA.^7^ In addition, **1b** displayed similar kinetic growth to palmitoleic acid (16:1 Δ^9^), a FA that has shown a cytotoxic effect on the growth of *S. aureus* by rapid membrane depolarization, disruption of the bacterial plasma membrane, and the release of critical proteins in the medium^26^. Thus, Figure 2 supports the idea that **1b** can disrupt the plasma membrane in *S. aureus*.

In order to better understand the mechanism of action of **1b**, we performed permeability studies involving CIMRSA XIII (Figure 3). Our results demonstrate that **1b** promotes fluorescein internalization inside the bacterial cell. This finding supports our hypothesis that membrane disruption occurs when MRSA is treated with **1b.**

When results in Figures 3 and 4 are analyzed, these suggest that VHFA provoke disruption of the *S. aureus* plasma membrane by inhibiting the expression of *norB*, which has repercussions on the biosynthesis of the NorB efflux pump. NorB is a surface protein that confers resistance to some antibiotics, such as hydrophilic fluoroquinolones, biocides, hydrophobic fluoroquinolones, and tetracycline.^27^ To some extent, **1b** and **2b** are internalizing the *S. aureus* plasma membrane, thus affecting the *norB* gene expression, as observed in Figure 4.

## 5. Conclusions

We further investigated how bromine and chlorine atoms affect the antibacterial activity of VHFA in CIMRSA. This study showed that VHFA containing a bromine atom is pivotal for their antibacterial activity against CIMRSA strains resistant to Cipro. Moreover, it was outlined that vinyl brominated FA can disrupt the *S. aureus* plasma membrane, likely by inhibiting the expression of the *norB* gene. This study will undoubtedly impact the understanding of the antibacterial properties of halogenated FA and highlight **1b** as a novel agent against nosocomial bacterial infections.

## Figures and Tables

**Table 1.** Antibacterial activity of **1a-1b** and **2a-2b** against several *S. aureus* strains and *E. coli*.

Compounds	MIC/GI_50_ ± SEM (μg/mL)							
	Bacterial strains							
	*E. coli* (ATCC 25922)	*S. aureus* (ATCC 29213)	*CIMRSA* XI	*CIMRSA* XII	*CIMRSA* XIII	*CIMRSA* XIV	*CIMRSA* XV	*CIMRSA* XVI
**1a**	>1000	>128	62.5/27.5 ± 1.2	250/95.1 ± 6.0	62.5/46.8 ± 1.2	62.5/30.4 ± 1.2	62.5/24.4 ± 1.1	62.5/42.5 ± 1.1
**1b**	>1000	62.5/45.4 ± 3.3	7.81/5.4 ± 1.1	15.6/10.0 ± 1.3	7.81/4.7 ± 1.9	15.6/9.3 ± 1.5	15.6/9.1 ± 1.1	7.81/4.1 ± 1.1
**2a**	>1000	15.6/6.6 ± 1.2	31.3/13.3 ± 1.2	62.5/47.7 ± 1.3	31.3/17.8 ± 1.2	31.3/6.7 ± 1.2	125/64.4 ± 1.1	31.3/12.1 ± 1.2
**2b**	>1000	125/86.5 ± 1.1	31.3/16.4 ± 1.1	62.5/54.0 ± 1.4	62.5/21.8 ± 1.4	125/72.4 ± 1.1	62.5/40.1 ± 1.1	62.5/22.6 ± 1.1
**2-HDA** ^b^	>1000	15.6/6.2 ± 0.9	0.49/0.30 ± 0.03	0.49/0.25 ± 0.03	0.24/0.16 ± 0.02	0.49/0.34 ± 0.03	0.49/0.27 ± 0.01	0.49/0.30 ± 0.03
**Cipro** ^b^	< 0.008	0.25/0.14 ± 0.01	7.8/5.7 ± 1.1	31.3/17.2 ± 1.0	62.5/30.4 ± 1.4	0.49/0.35 ± 0.04	7.8/4.7 ± 1.1	7.8/2.3 ± 1.1

a Experiments were performed in triplicate (N =3). GI_50_ values were obtained from dose-response curves.

b These results were obtained from Sanabria *et al*. 2020.^7^
